# Interaction between cytokine gene polymorphisms and the effect of physical exercise on clinical and inflammatory parameters in older women: study protocol for a randomized controlled trial

**DOI:** 10.1186/1745-6215-13-134

**Published:** 2012-08-08

**Authors:** Daniele S Pereira, Bárbara Z Queiroz, Elvis CC Mateo, Alexandra M Assumpção, Diogo C Felício, Aline S Miranda, Daniela MC Anjos, Fabianna Jesus-Moraleida, Rosângela C Dias, Danielle AG Pereira, Antônio L Teixeira, Leani SM Pereira

**Affiliations:** 1 Department of Physical Therapy, Graduate Program in Rehabilitation Sciences, School of Physical Education, Physical Therapy and Occupational Therapy, Universidade Federal de Minas Gerais, Belo Horizonte, MG, Brazil; 2 Department of Internal Medicine, School of Medicine, Universidade Federal de Minas Gerais (UFMG), Belo Horizonte, Brazil; 3 Departamento de Fisioterapia, Universidade Federal de Minas Gerais / UFMG, Av. Antônio Carlos, 6627, CEP 31270-901, Belo Horizonte, MG, Brazil

**Keywords:** Polymorphism, Cytokines, Older adult, Exercise, Physical performance, BDNF

## Abstract

**Background:**

Aging is associated with chronic low-grade inflammatory activity with an elevation of cytokine levels. An association between regular physical activity and reduction of blood levels of anti-inflammatory cytokines is demonstrated in the literature pointing to an anti-inflammatory effect related to exercise. However, there is no consensus regarding which type of exercise and which parameters are the most appropriate to influence inflammatory markers. Evidence indicates that the single nucleotide polymorphism (SNP) can influence the synthesis of those cytokines affecting their production.

**Methods/Design:**

The design of this study is a randomized controlled trial. The aim of this study is to investigate the interaction between the cytokine genes SNP and the effect of physical activity on older women. The main outcomes are: serum levels of sTNFR-1, sTNFR-2, interleukin (IL)-6, IL-10, measured by the ELISA method; genotyping of tumor necrosis factor- (TNF)-alpha (rs1800629), IL6 (rs1800795), IL10 (rs1800896) by the TaqMan Method (Applied Biosystems, Foster City, CA, USA); and physical performance assessed by Timed Up and Go and 10-Meter Walk Tests. Secondary outcomes include: Geriatric Depression Scale, Perceived Stress Scaleand aerobic capacity, assessed by the six-minute walk; and lower limb muscle strength, using an isokinetic dinamometer (Biodex Medical Systems, Inc., Shirley, NY,USA). Both exercise protocols will be performed three times a week for 10 weeks, 30 sessions in total.

**Discussion:**

Investigating the interaction between genetic factors and exercise effects of both protocols of exercise on the levels of inflammatory cytokine levels can contribute to guide clinical practice related to treatment and prevention of functional changes due to chronic inflammatory activity in older adults. This approach could develop new perspectives on preventive and treatment proposals in physical therapy and in the management of the older patient.

**Trial registration:**

(ReBEC) RBR9v9cwf

## Background

Aging is associated with a chronic low-grade inflammatory process characterized by a systemic elevation, from two to four times, of plasma levels of cytokines as interleukin (IL)-1, tumor necrosis factor alpha (TNF-α), IL-6, acute phase proteins, soluble TNF receptors (sTNFR) and IL-10, among others [1]. The balance between production and release of those cytokines has been related to the emergence or aggravation of chronic conditions related to aging, disability and increased mortality in olderadults[1,2]. High levels of cytokines are associated with a reduction of physical performance and muscle function [3-7]. The underlying mechanism by which these cytokines contribute to a functional deterioration in olderadults seems to be their catabolic effect, leading to a reduction in muscle mass and strength that are related to sarcopenia [1,8].

TNF-α is an early mediator of inflammation since it starts and coordinates the acute phase response and induces the production of a second wave of cytokine expression, such as IL-6, IL-8 and C-reactive protein [9]. It also stimulates the production of sTNFR that acts as its natural inhibitor; therefore, regulating its biological function. Since these receptors are more stable molecules than TNF-α in circulation, they are more reliable markers of plasma TNF-α levels, and hence of the inflammatory response [10]. A few authors argue that TNF-α is behind the age-related inflammatory changes [11,12], being associated with the development of insulin resistance and metabolic syndrome [13], and also with reduction of muscle mass and strength loss due to its catabolic action [14].

IL-6 is a cytokine that has both pro- and anti-inflammatory roles and is involved in controlling and coordinating inflammatory responses. It is produced by different cell types, which include the skeletal muscle cells [15]. On the other hand, IL-10 is an anti-inflammatory cytokine that is essential to inflammatory activity control and resolution that is triggered and sustained by other mediators [16]. The IL-10 inhibitory effect on IL-6 and TNF-α cytokines is well established in acute inflammatory processes [17], but not in chronic inflammation, such as we see during aging. The impact of physical exercises on their plasma levels is not known.

Differences seen in protein expression among people can occur as a result of functional genetic variations in the promoter area of these molecules gene [18]. The most common variations seen on human genomes are the single nucleotide polymorphism (SNP). Evidence points that SNPs, which are present in the genes of several molecules involved in inflammation, could affect their gene transcription and synthesis [18], modulating the inflammatory response severity. Some polymorphisms are associated with a greater production of inflammatory mediators. Thus, gene variations could explain in part the variability in the production of cytokines, and the greater liability of certain people to clinical conditions that are mediated by the elevation of these markers’ production, such as chronic conditions related to aging and longevity [19-21].

The expression of TNF-α, IL-6 and IL-10 is influenced by functional polymorphisms at their promoter areas. The polymorphisms in TNF-α (rs1800629), IL-6 (rs1800795) and IL-10 (rs1800896) have been associated with several acute and chronic diseases and with longevity as well [1,19,22]. For instance, Oberbach *et al.*[22] identified that changes in the IL-6 plasma levels in response to exercise were influenced by the -174 G/C polymorphism, suggesting that genetic factors related to cytokine production could be determinant to individual effects of the anti-inflammatory response promoted by exercise. However, literature presents contradictory results related to their activity and effects on plasma levels of these mediators, especially considering the older population. One possibility is that differences shown in the literature are due to interactions between lifestyle and gene factors, along with cultural and ethnic differences of the different studied samples.

Observational studies have pointed out that physically active people have lower concentrations of inflammatory cytokines, suggesting that regular exercise may alleviate the chronic inflammatory activity associated with aging [15,23,24]. Consistent evidence has demonstrated that physical activity induces an elevation of anti-inflammatory cytokine systemic levels [25-27], with the skeletal muscle tissue being indicated as an endocrine organ that produces and releases cytokines called miokines [15].

The production and release of IL-6 during physical activity seem to rely on factors such as type, intensity and duration of exercise [26]. Despite the possible benefits of physical exercise on chronic inflammatory process, information about the parameters of the ideal exercises for regulating cytokines have not been established [28-31], with research results being conflicting.

Therefore, the primary objective of this clinical trial is to investigate the existence of an interaction among TNF-α rs1800629, IL6 rs1800795, IL10 rs1800896 and gene polymorphisms with the effect of physical exercise in older women.

## Methods/Design

### Recruitment of participants

This study is a randomized controlled trial for which community-dwellingolder women will be recruited and randomly assigned to either one of the following physical activity protocol: muscle strengthening exercises (MSE) or aerobic exercises (AE). Participants will be recruited from the community through publicity in local newspapers and community centers for older persons. The study will be conducted at the Universidade Federal de Minas Gerais, Belo Horizonte, Brazil.

After the initial evaluation, participants will be randomized into either the MSE or AE group. Randomization will be performed by means of sealed brown envelopes without identification. The researcher responsible for the evaluations will be blinded to the participants’ group.

The study was approved by the Ethics Committee of Universidade Federal de Minas Gerais (ETIC 038/2010) and all volunteers will give their written informed consent to participate, according to the principles of the Helsinki Declaration (1964).

### Sample

#### Inclusion criteria

Community-dwellingolder women who are sedentary and aged 65 years or more will be included in the study. Sedentary older women are those who have not practiced any regular physical activity (that is, at least three times a week, 40 minutes minimum) in the last three months.

#### Exclusion criteria

Older people with cognitive impairment detectable by the Mini Mental State Examination [32], acute phase inflammatory disease, a history of cancer, current use of immunomodulatory medications, amputation or lower limb fracture in the last six months, presence of neurological sequelae or current participation in an alternative exercise program will be excluded from the study.

#### Baseline assessment

A standardized questionnaire will be applied by trained researchers to collect sample characteristics, socio-demographic data and information on the clinical condition of older adults.

#### Primary outcome measure

1. Plasma levels of TNF-α, sTNFR-1, sTNFR-2, IL-6, IL-10: 5 ml of blood will be withdrawn from the participants and immediately centrifuged at 1,500 rpm to obtain plasma. Aliquots will be removed in a laminar flow hood and stored at −80 °C. Plasma levels of cytokines will be determined by ELISA (enzyme-linked immunosorbent assay) method with high sensitivity kits (Quantikine®HS, R&D Systems, Minneapolis, MN, USA). According to the manufacturer the detection level of the method was 5 pg/ml for TNFRs, 0.15 pg/ml for IL-6 e 0.75 pg/ml for IL-10.

2. *Genotyping* – Blood samples will be obtained from all individuals in **ethylenediaminetetraacetic acid** (EDTA) anti-coagulant and genomic DNA will be extracted using a phenol-chloroform method from unfractionated whole blood, and stored at −20 °C. TaqMan genotyping assays will be obtained from Applied Biosystems, Inc. (Foster City, CA, USA). The assay identification code for each respective SNP is IL-10 (rs1800896), TNF (rs1800629). A customized assay will be used for the IL-6 SNP gene. All amplifications will be carried out in an ABI 7900HT thermal cycler (Applied Biosystems, Inc.) using TaqMan Genotyping Master Mix and following the manufacturer's recommended amplification conditions.

3. Physical Performance will be assessed by means of Timed Up and Go (TUG) [33] test and the 10-Meter Walk Test (10MWT) [34]. These tests will be used because they have demonstrated high reliability and are commonly used to assess function in older people. The TUG measures, in seconds, the time taken to stand up from a standard chair, walk a distance of three meters, turn, walk back to the chair and sit down. The TUG score demonstrates high inter-rater and intra-rater reliability (intraclass correlation coefficients (ICCs) 0.99 and 0.99 respectively) [35]. The 10MWT has good reliability (ICC = 0.78 e ICC = 0.93), and is a good marker for mobility and fall risk [36].

#### Secondary outcome measure

1. Muscle strength will be assessed using a Biodex® System 3 Pro isokinetic dynamometer (Biodex Medical Systems Inc., Shirley, NY,USA). This instrument has been accepted as the “gold standard” for assessment of muscular performance [37]. The muscle groups assessed will be the knee joint extensors and flexors of the dominant limb at angular speeds of 60° and 180° in concentric contractions, with 5 and 15 repetitions respectively, at intervals of 30 seconds between each velocity. All procedures will be performed according to the assessment protocol suggested by the manufacturer, such as positioning of the volunteer, calibration, correction for gravity, familiarization and strong verbal encouragement. Variables chosen for analysis are work normalized by body mass (%) at 60°, and Power at 180°.

2. Aerobic capacity, as measured by the 6-Minute Walk Test (6MWT). The 6MWT has been used in patients who have different disorders, such as neurological, cardiothoracic, infant or rheumatologic dysfunctions, and its correlation with maximum aerobic capacity is considered to be satisfactory [38,39]. Data related to systemic blood pressure, heart rate and subjective perception of effort using the Borg scale will be recorded.

3. Geriatric Depression Scale (GDS) is a screen for the presence of depression in older people. It comprises 15 individual questions; the cut-off-points will be 5/6 (non-case/case) [40,41].

4. The level of stress will be assessed by the 14-item Perceived Stress Scale validated for Brazilian older persons. This scale assesses three factors considered to be keys in the experience of stress: how the subject evaluates his life as unpredictable, uncontrollable and overloaded [42].

The salivary cortisol level will be measured as an objective measurement of stress [42]. The salivary cortisol level will be collected with cortisol specific Salivette tubes (Sarstedt.Salivette-swab). The dosage of the salivary levels will be performed using the ELISA method (*Salimetrics*).

### Intervention

The participants will be divided into two groups: strengthening exercises (SE) and aerobic exercises (AE) groups. Both groups will be submitted to a protocol lasting 10 weeks, 30 sessions in total, 3 times a week, under the direct supervision of a physical therapist.

### AE protocol

This protocol will consist of aerobic activities, including a 5-minute warm-up routine, 40 minutes of aerobic exercises – walking and free weight exercises for both upper and lower limbs, and a 5-minute cool down period, as recommended by the American College of Sports Medicine [43]. Heart rate will be maintained at 60% of age-predicted maximum heart rate during both warm-up and cool-down periods, and between 65% to 80% levels during the aerobic activity.

Blood pressure and heart rate will be measured at the beginning and at the end of every session. To ensure the safety of participants and to guarantee the proper training zone, each one of them will be monitored by a cardiac monitor.

### SE protocol

This program was based on a previous study (ISRCTN62824599) [44] developed by the Pain and Inflammation in Rehabilitation and Aging Studies Laboratory (Laboratório de Dor e Inflamação em Reabilitação e Estudos do Envelhecimento - LADIRE) research group.

The session will consist of a 10-minute walk, followed by stretching the rectus femoris, psoas, hamstrings and triceps surae muscles. Strengthening exercises will be performed for the following movements: hip flexion, abduction, adduction and extension; knee flexion and extension; mini-squat. The load, suitable for each participant, will be calculated by one repetition maximum (1RM) test. Participants will initiate the exercises at 50% of 1RM, adjusting the load after two weeks (seventh session) to 75% of 1RM. The RM will be recalculated for sessions 13 and 22, and the exercises will be performed at 75% of the newly established RM. Participants will be reassessed after 30 sessions.

### Sample size

Based on the multivariate linear regression analysis that will be performed in this study to explore the SNP influence on the effects of exercise on plasma levels of cytokines and onephysical performance, 140 older adults need to be included, considering the sample calculation of (10 x (K + 1), where K is the number of explanatory variables of the model. The explanatory variables are age, anthropometric measurements (Body Mass Index and Waist Circumference), physical activity level, presence of depression and stress levels (Perceived Stress Scale and salivary cortisol), TNF-α rs1800629 (AA + AG versus GG) , IL6 rs1800795 (GG versus CC + GC) , IL10 rs1800896 (GG versus AA + AG), genes polymorphisms.

### Statistical analyses

Insight into the sample characterization will be provided using descriptive statistics, including measures of central tendency (mean and median) and variability (range and standard deviation). Violation of Hardy-Weinberg equilibrium will be tested by the chi-square test.

The Kolmogorov-Smirnov test will be used to verify the normal distribution of data. The comparison for between and within groups data will be analyzed using an analysis of variance (ANOVA). *Post hoc* testing will be undertaken with LSD tests.

The multivariate linear regression analysis will be performed to explore the interaction and the effect of polymorphisms on the variables: plasma levels of cytokines and physical performance, considering the genotype of each investigated.

Statistical analysis will be performed using the *Statistical Package for Social Sciences* (SPSS Inc., Chicago, IL, USA), version 17.0, and α level will be set at .05.

## Discussion

The elevation of the plasma inflammatory cytokine levels has as its main consequences sarcopenia [6,18,19], reduction of function and independence of the older adult. We have previously demonstrated a weak to moderate negative correlation between high plasma levels of IL-6 and sTNFR-1, and reduced muscle strength and physical performance in older women [3,4].

Within this context, physical exercise has been presented as one of the most effective strategies to influence both an improvement in physical performance and a decrease in plasma levels of inflammatory markers in the older adult [7,24,29]. However, even though physical exercise has been widely performed in clinical practice, there is no consensus on which type of exercise and what clinical parameters are the most influential on the inflammatory markers. Thus, the results of this clinical trial could contribute to the standardization and management of clinical practice related to prevention and treatment of functional changes in the older adult.

A series of evidence suggests that the SNP could affect the production of cytokines, also influencing physical, cognitive and behavioral performance, and muscle strength in the olderadult [18,19,22]. We have recently investigated the effect of the -174 G/C polymorphism of the IL-6 gene on the plasma IL-6 levels in both community dwelling and institutionalized older women. Homozygotes women for the G allele showed high IL-6 levels, and an interaction between polymorphism and housing conditions (community/institution) was observed, with a higher effect of GG genotype on IL-6 levels in the institutionalized women [45].

The genes involved in the regulation of the chronic inflammatory process can contribute not only to the individual variability of cytokine production and physical performance in the older adult, but also to the response of these variables to physical exercise. Still, it is important to note that the evaluation of a single cytokine genotype without considering its interaction with other genotypes from different biological markers could lead us to misinterpretations, as their combined action could result in different effects. Therefore, analyzing and understanding the influence of genetic factors on the effects of different exercise protocols as proposed by this study may contribute to the development of new perspectives on preventive and therapeutic approaches in physical therapy and in general management of the older patient. This trial was designed to be reproducible in both research and clinical environments.

## Abbreviations

10MWT: 10-Meter Walk Test;1RM: one repetition maximum;6MWT: 6-Minute Walk Test;AE: aerobic exercises;EDTA: ethylenediaminetetraacetic acid;ELISA: Enzyme Linked Immunosorbant Assay;GDS: Geriatric Depression Scale;IL-10: interleukin-10;IL-6: interleukin-6;IL-8: interleukin-;MSE: muscle strengthening exercises;SNP: single nucleotide polymorphism;sTNFR: soluble TNF receptors;TNF-α: tumor necrosis factor alpha;TUG: Timed Up and Go.

## Competing interests

The authors declare that they have no competing interests.

## Authors’ contributions

DSP, LSMP, ALT and DAGP will be responsible for the conception and implementation of the study, as well as for its writing and final corrections. RCD will help in the implementation and supervision of the study. DMCA and BZQ will beresponsible for supervision of the intervention. AMA and DCF will be responsible for the evaluations. DSP, ECCM and BZQ will be responsible for the molecular genetic studies. ASM and DSP will be responsible for the immunoassays. DSP, DAGP and FJM will conduct the data analysis. All authors contributed to and approved the final manuscript.

## References

[B1] Krabbe KS Perdersen M Bruunsgaard H Inflammatory mediators in the elderly Exp Gerontol 2004 39 687 699 10.1016/j.exger.2004.01.009 15130663

[B2] Tiainen K Hurme M Hervonen A Luukkaala T Jylha M Inflammatory markers and physical performance among nonagenarians J Gerontol A Biol Sci Med Sci 2010 65 658 663 20421241 10.1093/gerona/glq056

[B3] Oliveira DM Narciso FM Santos ML Pereira DS Coelho FM Dias JM Pereira LS Muscle strength but not functional capacity is associated with plasma interleukin-6 levels of community-dwelling elderly women Braz J Med Biol Res 2008 41 1148 1153 10.1590/S0100-879X2008001200017 19148380

[B4] Pereira LSM Narciso FMS Oliveira DMG Coelho FM Souza DG Dias RC Correlation between manual muscle strength and interleukin-6 (IL-6) plasma levels in elderly community-dwelling women Arch Gerontol Geriatr 2009 48 313 316 10.1016/j.archger.2008.02.012 18462819

[B5] Brinkley TE Leng X Miller ME Kitzman DW Pahor M Berry MJ Marsh AP Kritchevsky SB Nicklas BJ Chronic inflammation is associated with low physical function in older adults across multiple comorbidities J Gerontol A Biol Sci Med Sci 2009 64 455 461 19196644 10.1093/gerona/gln038 PMC2657165

[B6] Ferrucci L Pennix BWJH Volpato S Harris TB Banden-Roche K Balfour J Leveille SG Fried LP Guralnik JM Change in muscle strength explains accelerated decline of physical function in older women with high interleukin-6 serum levels J Am Geriatr Soc 2002 50 1947 1954 10.1046/j.1532-5415.2002.50605.x 12473005

[B7] Coelho FM Pereira DS Lustosa LP Silva JP Dias JM Dias RC Queiroz BZ Teixeira AL Teixeira MM Pereira LS Physical therapy intervention (PTI) increases plasma brain-derived neurotrophic factor (BDNF) levels in non-frail and pre-frail elderly women Arch Gerontol Geriatr 2012 54 415 420 10.1016/j.archger.2011.05.014 21684022

[B8] Roubenoff R Physical activity, inflammation, and muscle loss Nutr Rev 2007 65 S208 S212 10.1301/nr.2007.dec.S208-S212 18240550

[B9] Makhatadze NJ Tumor necrosis factor locus: genetic organisation and biological implications Hum Immunol 1998 59 571 579 10.1016/S0198-8859(98)00056-1 9757913

[B10] Aderka D Engelmann H Shemer-Avni Y Hornik V Galil A Sarov B Wallach D Variation in serum levels of the soluble TNF receptors among healthy individuals Lymphokine Cytokine Res 1992 11 157 159 1327192

[B11] Roubenoff R Catabolism of aging: is it an inflammatory process? Curr Opin Clin Nutr Metab Care 2003 6 295 299 12690262 10.1097/01.mco.0000068965.34812.62

[B12] Bruunsgaard H Physical activity and modulation of systemic low-level inflammation J Leukoc Biol 2005 78 819 835 10.1189/jlb.0505247 16033812

[B13] Plomgaard P Nielsen AR Fischer CP Mortensen OH Broholm C Penkowa M Krogh-Madsen R Erikstrup C Lindegaard B Petersen AM Taudorf S Pedersen BK Associations between insulin resistance and TNF-alpha in plasma, skeletal muscle and adipose tissue in humans with and without type 2 diabetes Diabetologia 2007 50 2562 2571 10.1007/s00125-007-0834-6 17928988

[B14] Reid MB Li Y-P Tumor necrosis factor-α and muscle wasting: a cellular perspective Respir Res 2001 2 269 272 10.1186/rr67 11686894 PMC59514

[B15] Pedersen BK Febbraio MA Muscle as an endocrine organ: focus on muscle-derived interleukin-6 Physiol Rev 2008 88 1379 1406 10.1152/physrev.90100.2007 18923185

[B16] Moore KW Malefyt RW Coffman RL Interleukin-10 and the interleukin-10 receptor Annu Rev Immunol 2001 19 683 765 10.1146/annurev.immunol.19.1.683 11244051

[B17] Hempel L Korholz D Bonig H Interleukin-10 directly inhibits the interleukin-6 production in T-Cells Scand J Immunol 1995 41 462 466 10.1111/j.1365-3083.1995.tb03593.x 7725065

[B18] Maat MPM Bladbjerg EM Hjelmborg JB Bathum L Jespersen J Christensen K Genetic influence on inflammation variables in the elderly Arterioscler Thromb Vasc Biol 2004 24 2168 2173 10.1161/01.ATV.0000143856.01669.e7 15345506

[B19] Lio D Scola L Crivello A Colonna-Romano G Candore G Bonafe M Cavallone L Marchegiani F Olivieri F Franceschi C Caruso C Inflammation, genetics, and longevity: further studies on the protective effects in men of IL-10–1082 promoter SNP and its interaction with TNF-alpha −308 promoter SNP J Med Genet 2003 40 296 299 10.1136/jmg.40.4.296 12676903 PMC1735442

[B20] Stephens JW Hurel SJH Lowe GDO Rumley A Humphries SE Association between plasma IL-6, the IL-6–174G>C gene variant and the metabolic syndrome in type 2 diabetes mellitus Mol Genet Metab 2007 90 422 428 10.1016/j.ymgme.2006.10.004 17123852

[B21] Di Bona D Vasto S Capurso C Christiansen L Deiana L Franceschi C Hurme M Mocchegiani E Rea M Lio D Candore G Caruso C Effect of interleukin-6 polymorphisms on human longevity: a systematic review and meta-analysis Ageing Res Rev 2009 8 36 42 10.1016/j.arr.2008.09.001 18930842

[B22] Oberbach A Lehmann S Kirsch K Krist J Sonnabend M Linke A Tonjes A Stumvoll M Bluher M Kovacs P Long-term exercise training decreases interleukin-6 (IL-6) serum levels in subjects with impaired glucose tolerance: effect of the -174G/C variant in IL-6 gene Eur J Endocrinol 2008 159 129 136 10.1530/EJE-08-0220 18469018

[B23] Colbert LH Visser M Simonsick EM Tracy RP Newman A Kritchevsky SB Pahor M Taaffe DR Brach J Rubin S Physical activity, exercise, and inflammatory markers in older adults: findings from the health, aging and body composition study J Am Geriatr Soc 2004 52 1098 1104 10.1111/j.1532-5415.2004.52307.x 15209647

[B24] Kohut ML McCann DA Russell DW Konopka DN Cunnick JE Franke WD Castillo MC Reighard AE Vanderah E Aerobic exercise, but not flexibility/resistance exercise, reduces serum IL-18, CRP, and IL-6 independent of β-blockers, BMI, and psychosocial factors in older adults Brain Behav Immun 2006 20 201 209 10.1016/j.bbi.2005.12.002 16504463

[B25] Ostrowski K Schjerling P Pedersen BK Physical activity and plasma interleukin-6 in humans - effect of intensity of exercise Eur J Appl Physiol 2000 83 512 515 10.1007/s004210000312 11192058

[B26] Petersen AMW Pedersen BK The anti-inflammatory effect of exercise J Appl Physiol 2005 98 1154 1162 10.1152/japplphysiol.00164.2004 15772055

[B27] Mathur N Pedersen BK Exercise as a mean to control low-grade systemic inflammation Mediators Inflamm 2008 2008 109502 19148295 10.1155/2008/109502 PMC2615833

[B28] Nicklas BJ Hsu FC Brinkley TJ Church T Goodpaster BH Kritchevsky SB Pahor M Exercise training and plasma C-reactive protein and interleukin-6 in elderly people J Am Geriatr Soc 2008 56 2045 2052 10.1111/j.1532-5415.2008.01994.x 19016938 PMC2683336

[B29] Greiwe JS Cheng B Rubin DC Yarasheski KE Semenkovich CF Resistance exercise decreases skeletal muscle tumor necrosis factor a in frail elderly humans FASEB J 2001 15 475 482 10.1096/fj.00-0274com 11156963

[B30] Nicklas BJ Ambrosius W Messier SP Miller GD Penninx BW Loeser RF Palla S Bleecker E Pahor M Diet-induced weight loss, exercise, and chronic inflammation in older, obese adults: a randomized controlled clinical trial Am J Clin Nutr 2004 79 544 551 15051595 10.1093/ajcn/79.4.544

[B31] Phillips MD Flynn MG McFarlin BK Stewart LK Timmerman KL Resistance training at eight-repetition maximum reduces the inflammatory milieu in elderly women Med Sci Sports Exerc 2010 42 314 325 19927028 10.1249/MSS.0b013e3181b11ab7

[B32] Folstein MF Folstein SE McHugh PR Minimental state. A practical method for grading the cognitive status of patients for the clinician J Psychiatr Res 1975 12 189 198 10.1016/0022-3956(75)90026-6 1202204

[B33] Podsiadlo D Richardson S The timed up & go: a test of basic functional mobility for frail elderly persons J Am Geriatr Soc 1991 39 142 148 1991946 10.1111/j.1532-5415.1991.tb01616.x

[B34] Shinkai S Watanabe S Kumagai S Fujiwara Y Amano H Yoshida H Ishizaki T Yukawa H Suzuki T Shibata H Walking speed as a good predictor for the onset of functional dependence in a Japanese rural community population Age Ageing 2000 29 441 446 10.1093/ageing/29.5.441 11108417

[B35] Bohannon RW Reference values for the timed up and go test: a descriptive meta-analysis J Geriatr Phys Ther 2006 29 64 68 10.1519/00139143-200608000-00004 16914068

[B36] VanSwearingen JM Brach JS Making geriatric assessment work: selecting useful measures Phys Ther 2001 81 1233 1252 11380279

[B37] Drouin JM Valovich-mcLeod TC Shultz SJ Gansneder BM Perrin DH Reliability and validity of the Biodex system 3 pro isokinetic dynamometer velocity, torque and position measurements Eur J Appl Physiol 2004 91 22 29 10.1007/s00421-003-0933-0 14508689

[B38] Enright PL The six-minute walk test Respir Care 2003 48 783 785 12890299

[B39] Solway S Brooks D Lacasse Y Thomas S A qualitative systematic overview of the measurement properties of functional walk tests used in the cardiorespiratory domain Chest 2001 119 256 270 10.1378/chest.119.1.256 11157613

[B40] Yesavage JA Brink TL Rose TL Lum O Huang V Adey M Development and validation of a geriatric depression screening scale: a preliminary report J Psychiatr Res 1982 17 37 49 10.1016/0022-3956(82)90033-4 7183759

[B41] Almeida OP Almeida SA Confiabilidade da versão brasileira da Escala de Depressão em Geriatria (GDS) versão reduzida Arq Neuropsiquiatr 1999 57 421 426 10.1590/S0004-282X1999000300013 10450349

[B42] Luft CD Sanches SO Mazo GZ Andrade A Brazilian version of the Perceived Stress Scale: translation and validation for the elderly Rev Saude Publica 2007 41 606 615 17589759 10.1590/s0034-89102007000400015

[B43] Chodzko-Zajko WJ Proctor DN Fiatarone Singh MA Minson CT Nigg CR Salem GJ Skinner JS American College of Sports Medicine position stand. Exercise and physical activity for older adults Med Sci Sports Exerc 2009 41 1510 1530 10.1249/MSS.0b013e3181a0c95c 19516148

[B44] Lustosa LP Coelho FM Silva JP Pereira DS Parentoni AN Dias JM Dias RC Pereira LS The effects of a muscle resistance program on the functional capacity, knee extensor muscle strength and plasma levels of IL-6 and TNF-alpha in pre-frail elderly women: a randomized crossover clinical trial--a study protoco Trials 2010 11 82 10.1186/1745-6215-11-82 20667082 PMC2919525

[B45] Pereira DS Garcia DM Narciso FM Santos ML Dias JM Queiroz BZ Souza ER Nobrega OT Pereira LS Effects of 174G/C polymorphism in the promoter region of the interleukin-6 gene on plasma IL-6 levels and muscle strength in elderly women Braz J Med Biol Res 2011 44 123 129 10.1590/S0100-879X2010007500152 21180882

